# Changes in real-world dispensing of ADHD stimulants in youth from 2019 to 2021 in California

**DOI:** 10.3389/fpubh.2024.1302144

**Published:** 2024-03-05

**Authors:** Anika Patel, Rishikesh Chavan, Cyril Rakovski, Richard Beuttler, Sun Yang

**Affiliations:** ^1^Department of Pharmacy Practice, Chapman University School of Pharmacy, Irvine, CA, United States; ^2^Hyundai Cancer Institute, CHOC Children’s Hospital, Orange, CA, United States; ^3^Schmid College of Science of Technology, Chapman University, Orange, CA, United States; ^4^Chapman University School of Pharmacy, Irvine, CA, United States

**Keywords:** ADHD, stimulants, pandemic, disparities, controlled substances, pediatric, youth, socioeconomic impact

## Abstract

**Introduction:**

Attention-deficit/hyperactivity disorder (ADHD) is one of the most common pediatric neurobehavioral disorders in the U.S. Stimulants, classified as controlled substances, are commonly used for ADHD management. We conducted an analysis of real-world stimulants dispensing data to evaluate the pandemic’s impact on young patients (≤ 26 years) in California.

**Methods:**

Annual prevalence of patients on stimulants *per capita* across various California counties from 2019 and 2021 were analyzed and further compared across different years, sexes, and age groups. New patients initiating simulants therapy were also examined. A case study was conducted to determine the impact of socioeconomic status on patient prevalence within different quintiles in Los Angeles County using patient zip codes. Logistic regression analysis using *R Project* was employed to determine demographic factors associated with concurrent use of stimulants with other controlled substances.

**Results:**

There was a notable reduction in prevalence of patients ≤26 years old on stimulants during and after the pandemic per 100,000 people (777 in 2019; 743 in 2020; 751 in 2021). These decreases were more evident among the elementary and adolescent age groups. The most prevalent age group on stimulants were adolescents (12–17 years) irrespective of the pandemic. A significant rise in the number of female patients using stimulants was observed, increasing from 107,957 (35.2%) in 2019 to 121,241 (41.1%) in 2021. New patients initiating stimulants rose from 102,754 in 2020 to 106,660 in 2021, with 33.2% being young adults. In Los Angeles County, there was an increasing trend in patient prevalence from Q1 to Q5 income quintiles among patients ≥6 years. Consistently each year, the highest average income quintile exhibited the highest *per capita* prevalence. Age was associated with higher risk of concurrent use of benzodiazepines (OR, 1.198 [95% CI, 1.195–1.201], *p* < 0.0001) and opioids (OR, 1.132 [95% CI, 1.130–1.134], *p* < 0.0001) with stimulants.

**Discussion:**

Our study provides real-world information on dispensing of ADHD stimulants in California youth from 2019 to 2021. The results underscore the importance of optimizing evidence-based ADHD management in pediatric patients and young adults to mitigate disparities in the use of stimulants.

## 1 Introduction

The mental health of pediatric patients has attracted more attention in recent years. From 2009 to 2015, mental health-related emergency department visits increased by 56.4% for pediatric patients (1). Attention-deficit/hyperactivity disorder (ADHD) is a chronic neurological disorder that tends to manifest in early childhood with symptoms including hyperactivity, impulsivity, and difficulty in maintaining attention that are debilitating in many aspects of life (2). There has been an increasing trend in the diagnosis of ADHD over the years, with about 6 million children between 3 and 17 years old diagnosed in the United States from 2016 to 2019 (3, 4). In 2011, 6.7% of children in California (CA) were diagnosed with ADHD (4, 5).

The unprecedented COVID-19 pandemic had a noticeable impact on mental health and neurological-related disorders throughout the pediatric population globally (6). Subsequent changes in daily routine and school studies in response to the stay-at-home orders and limited interpersonal interactions, presented a strain on this vulnerable population. Behavioral changes and increased ADHD symptoms were reported in pediatric patients with ADHD (7, 8). In California, school districts began closing classrooms in March of 2020 followed by periodical state-wide stay-at-home orders and restrictions that lasted until June of 2021, at which time California reopened without capacity restrictions or distancing requirements.

As recommended by the American Academy of Pediatrics (AAP) clinical practice guideline (2019), pharmacotherapy and behavioral interventions constitute the treatment and management of ADHD (9). Pharmaceutical treatment includes non-stimulants and stimulant medications, specifically methylphenidate and amphetamines, which are Schedule II controlled substances approved by FDA for ages 3 years and up. Given their safety profile and efficacy in reducing ADHD symptoms, stimulants alone or in combination with behavioral therapy remain the mainstay of ADHD pharmacotherapy for pediatric patients (9, 10).

The Controlled Substance Utilization Review and Evaluation System (CURES) is a database of Schedule II-V controlled substance prescriptions dispensed in CA. State law mandates that each controlled substance prescription should be reported to CURES by the dispensing authority within one working day of the prescription being released to the patient or patient’s representative. Using the CURES de-identified database, we analyzed changes in the prescribing and dispensing of ADHD stimulants during and after the pandemic in pediatric and young adult patients (≤ 26 years). In addition, analysis was conducted to assess potential demographic, pharmacotherapeutic, and geographic characteristics associated with ADHD stimulant use in CA. Moreover, we compared concurrent use of stimulants with other controlled substances among youths from 2019 to 2021.

## 2 Methods

### 2.1 Data preparation

De-identified individual-level CURES data was obtained from the Department of Justice (DOJ) for the 2019–2021 period. This database contains 22 variables, including Patient ID, Pharmacy ID, Prescriber ID, Patient Birth Year, Gender, Patient City, Patient State, Patient Zip Code, Prescriber Zip Code, Pharmacy Zip Code, Product Name, NDC Number, Drug Form, Strength, Quantity, Days of Supply, Date Filled, Drug Refill Number, Drug Refill Authorized Number, Payment Codes, License Board, and License Type.

Institutional Review Board (IRB) exemption was obtained through Chapman University. The database was narrowed down to only include prescription entries of patients between 0 and 26 years old who were dispensed an ADHD stimulant. For the purposes of this study, 2019 was defined as pre-pandemic, 2020 as pandemic, and 2021 as post-pandemic. The age ranges were defined as infancy (0–2 years), preschool (3–5 years), elementary school (6–11 years), adolescent (12–17 years), late adolescent (18–21 years), and young adult (22–26 years) and were assigned based on each year’s data individually.

### 2.2 Statistical analysis

The dataset was prepared, formatted, and analyzed using the *R Project* for Statistical Computing (Version 4.2.1). Descriptive statistics were used to assess the changes in ADHD stimulants dispensed in youth in CA from 2019 to 2021.

ADHD stimulant dispensing was further analyzed geographically and socioeconomically based on patient’s zip codes. The geographic prevalence of patients dispensed ADHD stimulants was compared by California counties. Los Angeles (LA) County presented as a case study to analyze possible relationships between socioeconomic status and patient prevalence given the large number of patients on stimulants and vast diversity in socioeconomic status. The median household incomes by LA County zip codes were obtained from the United States Census Bureau. Each patient was placed into different income quintiles based on the average household income of their zip code. Patients among different quintiles were further categorized by age group. *Per capita* values were calculated using the U.S. Census Bureau population counts per California County and Los Angeles County zip code to estimate prevalence. The *R* packages “*usmap*” and “*ggplot2*” were used to produce heat maps of the stimulants dispensed from 2019 to 2021 (11, 12).

Using the unique patient identification numbers (ID), new patients were defined as those who had no stimulant prescriptions in the previous year. Analysis of new ADHD patients was limited to 2020 and 2021, due to the 2018 dataset being incomplete as mandatory use of CURES became effective in October of 2018 in California. Further analysis was done on patients who had prescribed other controlled substances in addition to stimulants, such as benzodiazepines or opioids, as identified by the same patient ID. A mixed effects logistic regression analysis was conducted to control for repeated entries of the same patients across the years. The “*car*” and “*lme4*” packages were used to determine if patient demographic factors were associated with concurrent use of other controlled substances with stimulants (13, 14). Probability curves were generated from logistic regression analysis using the “*ggplot2*” package. A two-sided *p* ≤ 0.05 was considered statistically significant.

## 3 Results

### 3.1 Trends of patients on ADHD stimulants throughout the pandemic

The annual prevalence of patients (0–26 years old) on ADHD stimulants per 100,000 people in California decreased from 777 in 2019 to 743 in 2020 and 751 in 2021 (Table 1, Supplementary Figure S1). In 2019, Fresno County showed the highest prevalence (916 per 100,000 people), which markedly dropped to 830 in 2020 and 766 in 2021. Most of the top ten counties in CA by the number of patients showed a downward trend in prevalence, except Santa Clara County and San Diego County, which were even higher in 2021 than in 2019.

**Table 1:** Prevalence of patients (≤ 26 years) on ADHD stimulants per 100,000 people by county in California (2019–2021).

	2019		2020		2021	
	Patients 0–26 years	Per 100,000 people	Patients 0–26 years	Per 100,000 people	Patients 0–26 years	Per 100,000 people
California	306,998	777	293,430	743	294,744	751
Los Angeles	64,915	647	61,632	617	61,115	622
San Diego	26,846	804	25,990	788	27,041	823
Orange	27,991	881	27,258	856	27,433	866
Riverside	16,015	648	14,837	612	14,513	590
San Bernardino	13,396	615	12,104	555	11,839	539
Santa Clara	12,326	639	12,331	639	12,812	680
Alameda	10,929	654	10,428	621	10,738	651
Sacramento	13,820	890	12,969	818	12,783	805
Contra Costa	9,960	863	9,521	817	9,528	820
Fresno	9,148	916	8,373	830	7,763	766

Population counts by California County were obtained from the United States Census Bureau. The top ten counties by number of patients are summarized in the table.

The sex distribution of patients on ADHD stimulants remained consistent with male predominance throughout the years. Of note, during the study period, the percentage of male patients trended downwards, decreasing from 64.8% (2019) to 62.6% (2020) and 58.8% (2021) (Table 2). In addition, among the late adolescent and young adult populations, a more equal sex distribution was observed (Supplementary Figure S2).

**Table 2:** Demographic characteristics of the total and new patients ≤ 26 years old on ADHD stimulants in CA.

	Total patients (≤ 26 years)			New patients* (≤ 26 years)	
	2019	2020	2021	2020	2021
	(*n* = 306,998)	(*n* = 293,430)	(*n* = 294,744)	(*n* = 102,754)	(*n* = 106,660)
	No. (%)	No. (%)	No. (%)	No. (%)	No. (%)
Gender					
Male	198,987 (64.8)	183,663 (62.6)	173,393 (58.8)	58,114 (56.6)	54,486 (51.1)
Female	107,957 (35.2)	109,717 (37.4)	121,241 (41.1)	44,612 (43.4)	52,090 (48.8)
Unknown	54 (< 0.1)	50 (< 0.1)	110 (< 0.1)	28 (< 0.1)	84 (< 0.1)
Age groups					
Infancy (0–2)	12 (< 0.1)	9 (< 0.1)	10 (< 0.1)	9 (< 0.1)	8 (< 0.1)
Preschool (3–5)	1856 (0.6)	1,423 (0.5)	1,436 (0.5)	1,227 (1.2)	1,289 (1.2)
Elementary (6–11)	78,676 (25.6)	69,901 (23.8)	61,688 (20.9)	27,089 (26.4)	23,421 (22.0)
Adolescent (12–17)	113,962 (37.1)	107,027 (36.5)	101,898 (34.6)	28,098 (27.3)	27,221 (25.5)
Late adolescent (18–21)	48,086 (15.7)	48,372 (16.5)	53,409 (18.1)	16,668 (16.2)	19,350 (18.1)
Young adults (22–26)	64,406 (21.0)	66,698 (22.7)	76,303 (25.9)	29,663 (28.9)	35,371 (33.2)

* Patients with no ADHD stimulant prescriptions in the previous year, but with at least one prescription in the given year were defined as new patients.

In 2019, the most prevalent age group on ADHD stimulants was adolescents (12–17 years, 37.1%), followed by elementary (6–11 years, 25.6%), and young adults (22–26 years, 21%). However, in 2021, young adults surpassed the elementary age group and become the second highest group on ADHD stimulants, increasing from 64,406 to 76,303 (25.9%). The number of late adolescent patients also increased from 48,086 (15.7%, 2019) to 53,409 (18.1%, 2021). On the contrary, the elementary age patients markedly dropped from 78,676 (2019) to 61,688 (2021) (Table 2). A reduction of adolescent patients was also noted, while still remaining predominant compared to all other age groups (101,898, 34.6% in 2021).

### 3.2 New patients on ADHD stimulants

The total number of new patients identified in 2021 was 106,660, increasing from 102,754 in 2020 (Table 2). The majority of new patients initiating stimulant therapy were young adults (33.2%) followed by adolescents (25.5%), consistent with the year of 2020 (28.9 and 27.3%, respectively). Among new patients, the proportion of females increased from 43.3% in 2020 to 48.8% in 2021, while there was a substantial decrease in the number of new elementary and adolescent patients in 2021. Notable increases of new patients were most evident among late adolescents and young adults.

### 3.3 Disparities in ADHD stimulant dispensing in Los Angeles (LA) County

**Figure 1:**
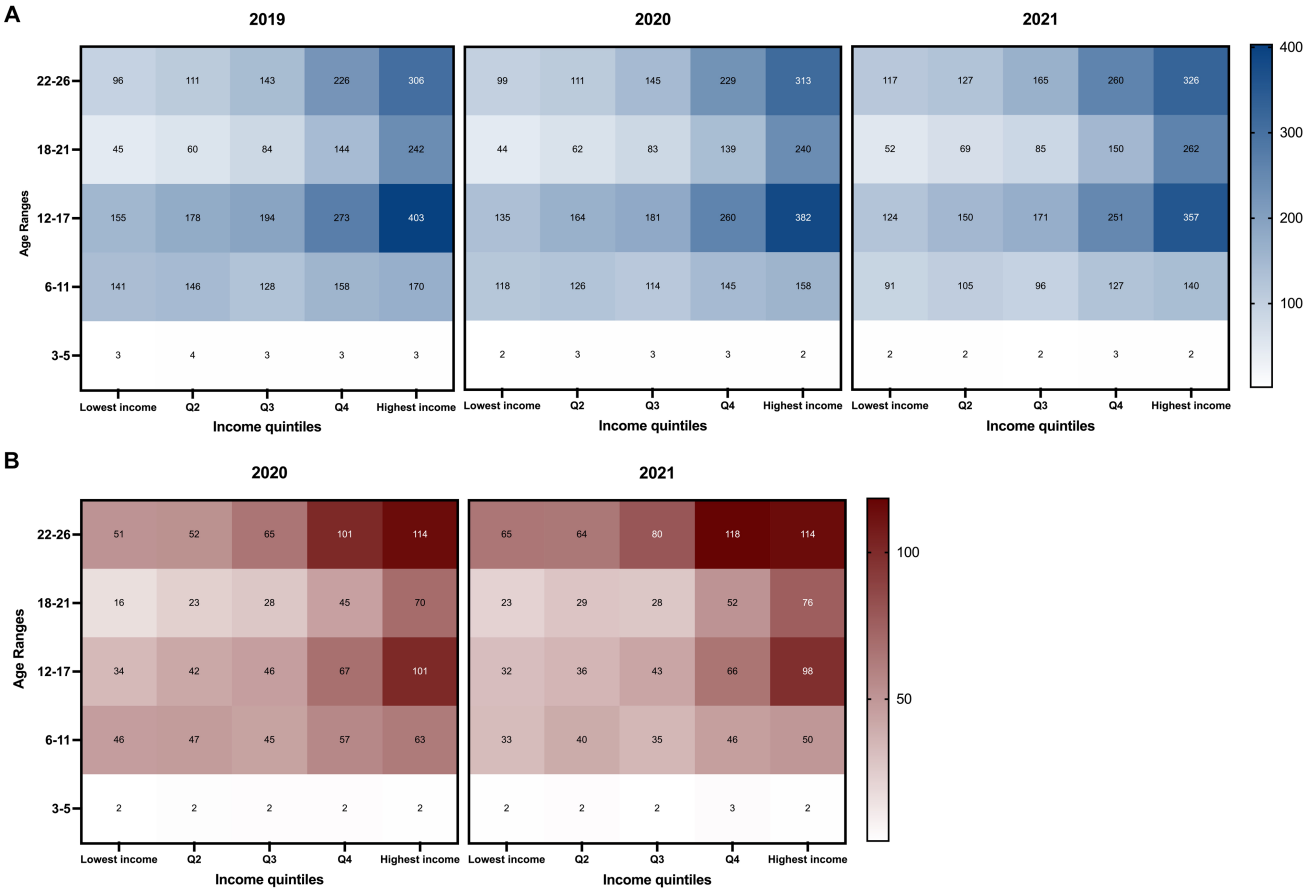
Trends in annual prevalence of patients (≤ 26 years) on ADHD stimulants by patient age group and annual household income from 2019 to 2021 in Los Angeles County, California. The x-axis represents income quintiles determined by the average household income based on Los Angeles County zip codes obtained from the U.S. Census Bureau. The y-axis represents different age groups. Patient prevalence was tabulated by the total population of each quintile based on zip code quintile designation. The color scales on the right side indicate the number of patients per 100,000 people. The darkest colors represent the highest value range. Patients between the ages of 0–2 years had an annual prevalence of less than 0.1 for all quintile classifications and was not included in the figure. Prevalence of patients **(A)** and new patients **(B)** (≤ 26 years) on ADHD stimulant medications by age group and patient zip codes.

LA County had the highest number of patients (0–26 years old) on stimulant prescriptions in CA each year (Table 1) with a population representing a broad range of household incomes. As shown in Figure 1A, there is a notable increasing trend in patient prevalence per 100,000 people from Q1 to Q5 among the elementary, adolescent, late adolescent, and young adult populations. This increase in patient prevalence by quintile is also evident in new ADHD patients in 2020 and 2021 (Figure 1B). Consistently each year, the Q5 income quintile showed the highest prevalence *per capita* among all age groups ≥6 years, either in all ADHD patients or among the new patients, independent of the pandemic.

Between 2019 and 2021, the prevalence of the Q1 income quintile decreased among elementary and adolescent age groups, while it rose among late adolescents and young adults. This trend is also observed in other income quintiles, which may be a notable shift attributed to the pandemic. In LA county, the prevalence of adolescents in the Q5 quintile has decreased over the years (2019: 403; 2020: 382; and 2021: 357) but remains significantly higher (2.88-times) than that of Q1 in 2021. The rise in prevalence among young adults was noted across all income quintiles from 2019 to 2021 (Figure 1A).

For new patients, the age group with the highest *per capita* number is predominantly the young adult population (22–26 years) in the Q4 and Q5 quintiles, followed by the adolescents in the Q5 income quintile (Figure 1B).

### 3.4 Use of stimulants with other controlled substances

The total number of patients (≤ 26 years) concurrently using stimulants with benzodiazepines decreased from 14,419 in 2019 to 12,813 in 2020 and 12,799 in 2021 (Supplementary Figure S3). However, in all three years, a higher percentage of females were observed to use stimulants in combination with benzodiazepines compared to males, and this trend showed an annual increase (2019: 56.1%, 2020: 59.3%, and 2021: 62.4%).

The total number of patients using opioids with stimulants was 15,341, 13,635, and 15,257 in 2019, 2020, and 2021, respectively (Supplementary Figure S4). The percentage of female patients was lower than that of males in 2019 (46.2%) but become the predominant group by 2021 (53.4%).

**Figure 2:**
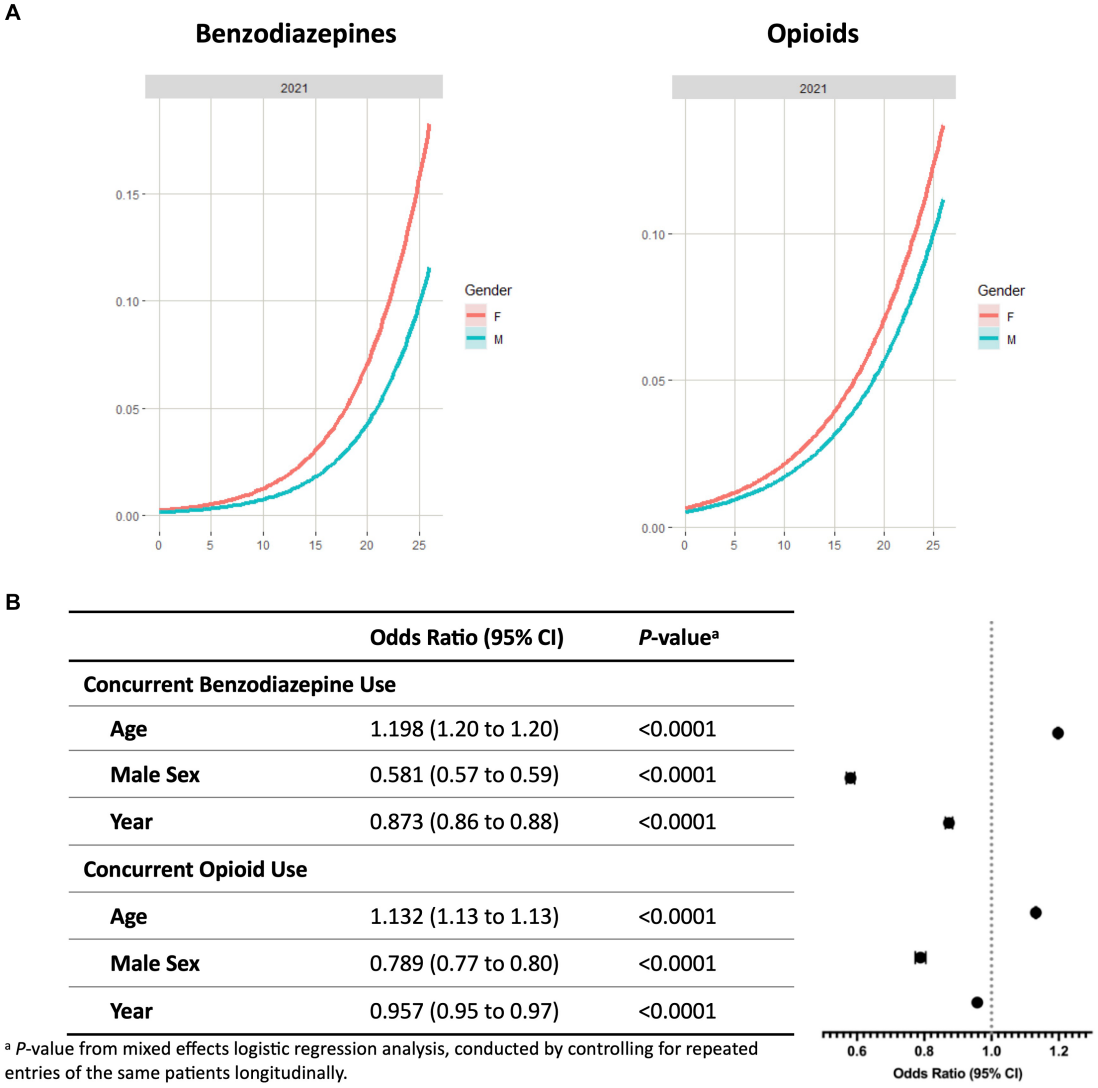
Concurrent benzodiazepine and opioid use with ADHD stimulants among patients ≤26 years in California. **(A)** Probability curves show the probability of concurrent controlled substance use with stimulants based on patient age in 2021. Females (red line) showed an increased risk at any age for concurrent use of benzodiazepines or opioids with stimulants in comparison to males (blue line). Similar trends were seen in 2019 and 2020 (Supplementary Figure S6). **(B)** Covariates including year, sex, and age were analyzed to determine odds ratios of concurrent use of controlled substances with stimulants. ^a^*p*-value from mixed effects logistic regression analysis, conducted by controlling for repeated entries of the same patients longitudinally.

As shown in Figure 2A, the probability of concurrent use of benzodiazepines or opioids with stimulants increased with age in CA independent of the year studied (Figure 2A; Supplementary Figure S5). The highest prevalence of concurrent use was observed in young adults (22–26 years) for all three years of the study, followed by late adolescents (18–21 years) and adolescents (12–17 years). Among the young adult patients, 9.3% were on a stimulant and a benzodiazepine, 6.1% were on a stimulant and an opioid, and 2.2% were on all three classes of controlled substances.

Mixed effects logistic regression analysis showed that patient age is associated with higher risk of concurrent use of stimulants with benzodiazepines (OR, 1.198 [95% CI, 1.195 to 1.201], *p* < 0.0001) or opioids (OR, 1.132 [95% CI, 1.130 to 1.134], *p* < 0.0001) (Figure 2B). Covariates associated with reduced risk include the study year and male sex. Females exhibited a higher probability of concurrent use from 0 to 26 years old. For benzodiazepines, the ORs of the study year and male sex were 0.873 (95% CI, 0.862 to 0.884, *p* < 0.0001) and 0.581 (95% CI, 0.568 to 0.593, *p* < 0.0001), respectively. Similar patterns were observed in patients on concurrent use of opioids and stimulants (OR of study year, 0.957 [95% CI 0.946 to 0.969], *p* < 0.0001; OR of male sex, 0.789 [95% CI 0.773 to 0.805], *p* < 0.0001).

### 3.5 ADHD stimulant prescription and dispensing trends from 2019 to 2021

**Figure 3:**
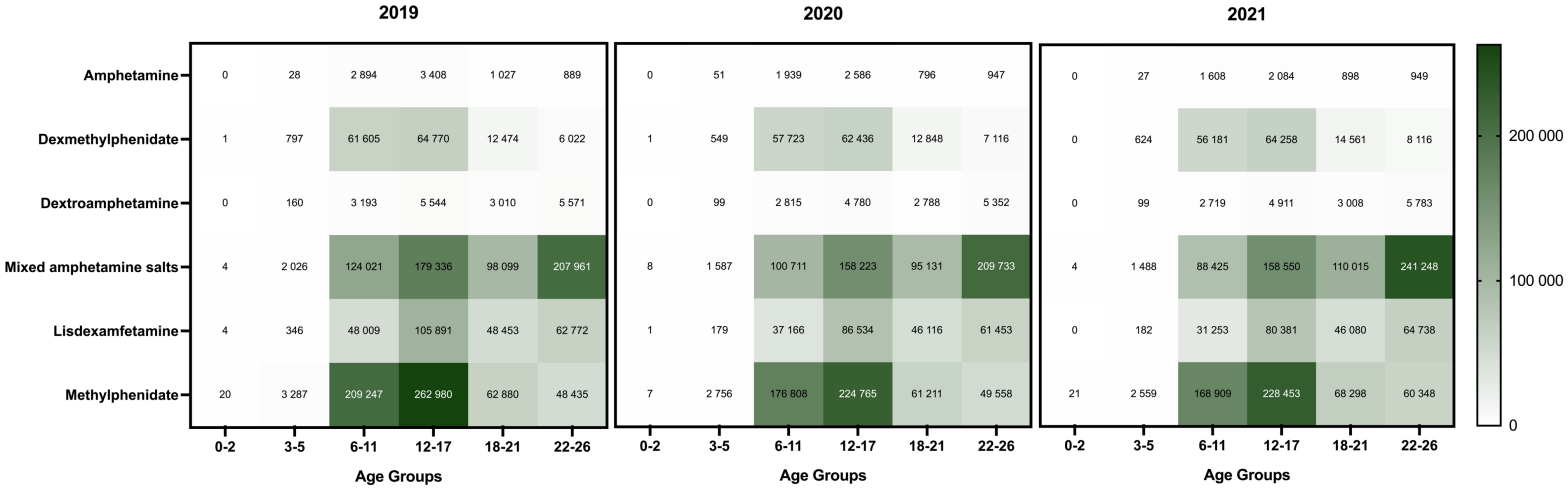
Distribution of ADHD stimulant classes prescribed to patients (≤26 years) in California (2019–2021). The color scale on the right side indicates the total number of dispensed stimulant prescriptions of each drug class by age group. The darkest color represents the highest value range.

The most predominant stimulant prescriptions dispensed annually were the mixed amphetamine salts and methylphenidate, with the highest number of prescriptions dispensed in 2019 (Supplementary Figure S6). Methylphenidate was primarily prescribed to patients aged 6–17, while among young adults, the top stimulants dispensed were mixed amphetamine salts (Figure 3). Lisdexamfetamine, the prodrug formulation with lower abuse potential, was predominantly prescribed to adolescents, and there was a notable decrease from 2019 to 2021. The profile of stimulant classes prescribed in each age group remained consistent between 2019 and 2021, regardless of the pandemic.

## 4 Discussion

Emerging evidence has demonstrated the negative impact of the COVID-19 pandemic on mental health in children, particularly among adolescents (15–17). During this time, children with previously diagnosed ADHD presented with more behavioral issues, more ADHD-related symptoms such as emotional difficulties and conduct behaviors and decreased psychological well-being (18). Major challenges of ADHD management were exacerbated by the pandemic, including making an accurate diagnosis, identifying psychiatric comorbidities, effective communication between key participants, misuse of stimulants, and medication access (19). Patient access was also hindered by unemployment, lack of insurance or coverage, and drug shortages. The increased use of telemedicine helps physicians observe children in their natural environment and offers more consistent follow up to optimize patient care (19). However, given the potential for abuse, the Drug Enforcement Administration is taking steps to implement necessary safeguards restricting accessing ADHD stimulants through telemedicine (20).

The overall number of ADHD patients ≤26 years decreased in 2020 compared to pre-pandemic, with a marked drop among the elementary and adolescent populations. Consistently, studies by other groups demonstrated a significant decrease in psychiatric visits and pharmacotherapy in the child and adolescent populations in 2020 in comparison to 2019 (21). Furthermore, a 70% decrease in referrals for school-based behavioral health services was noted from 2018 to 2020 (22). Thus, the decrease in patient prevalence observed in our study can potentially be attributed to a combination of educational and healthcare-related factors.

With analyzing the CURES database, we observed that the general population characteristics of patients receiving ADHD stimulant therapy remained relatively consistent during and following the pandemic. There were significantly more male patients compared to females, which may be explained by the higher reported rates of ADHD diagnosis in males (4). However, female patients on stimulants notably increased by 6.7% in 2021 compared to pre-pandemic, while there was a decreasing trend in the percentage of ADHD stimulants prescribed to males from 2019 to 2021. An earlier behavioral questionnaire analysis revealed that the mental wellbeing of girls during the COVID-19 pandemic declined more than that of boys (23). In addition, studies have shown increases in mental health-related outpatient physician visits, emergency department visits and psychotropic drug prescriptions dispensed during the pandemic, with significant increases shown in the 10–19-year-old and female populations (24). In 2020 and 2021, 43.4 and 48.8% of new patients starting stimulant therapy were female, respectively, which were higher than the percentages of total female patients ≤26 years (37.4 and 41.1%). Consistently, previous studies showed that the diagnosis rates of female youths were trending upwards at a higher rate than that of male youths, increasing by 22% in females and 17% in males from 2008 to 2013 (25). An increase of 29% in starting ADHD pharmacotherapy was also reported in females compared to 10% in males (25).

A national survey analysis showed that pediatric stimulant use has been constantly increasing from 1996 to 2008, primarily because of greater use in adolescents (26). In California, our study demonstrated that the population with the highest number of patients on stimulants in 2019–2021 were adolescents. The number of late adolescent and young adult patients notably increased in 2021 in comparison to 2019. Adolescents, especially the ones with pre-existing mental health disorders, were vulnerable to the negative impacts of the pandemic on mental health (6). Significant challenges encountered during the pandemic included online learning, boredom, and social isolation due to lack of routine and greater difficulty concentrating (27, 28). During the pandemic, adolescents with ADHD experienced an increase in external symptoms independent of drug holidays, and their quality and duration of sleep were also impacted (29).

Additionally, among the new patients initiating stimulant therapy during and following the pandemic, the predominant age group were young adults aged 22–26 years old. The high prevalence of stimulant use among adolescents and young adults raises concerns about diversion and the general misuse of controlled substances (30). As of May 2023, the FDA has required new prescription stimulant labeling emphasizing the risks of misuse, abuse, addiction, and overdose with this class of medications (31). Studies showed that about 15% of adolescents and young adults with ADHD are estimated to have a concurrent substance use disorder (32–34). Particularly, the 18–25-year-old population has been shown to have the highest level of misuse of these medications in comparison to other age groups (30).

Our study demonstrated that the probability of concurrent use of these controlled substances increases with age and is consistently higher in female youth in all age groups. Healthcare providers need to consider possible comorbid conditions such as anxiety, depression, bipolar and personality disorders when prescribing. This is especially important for female patients, as the prevalence of comorbid conditions is higher in females compared to males (35). A previous study analyzed opioid and other controlled substance usage from 2011 to 2015 in California and found notable disparities in opioid overdosing, and the overdose deaths were highly concentrated in lower-income and mostly white areas (36). Furthermore, their study demonstrated that females were utilizing opioids and benzodiazepines at higher rates than males, which is consistent with our observations that females have a higher risk in concurrent use of benzodiazepines or opioids with stimulants. Our odds ratios for the year covariate reflected a decrease from year to year for the number of patients on concurrent stimulant and benzodiazepines/opioids. However, a larger decrease in number of patients occurs between 2019 and 2020 during the pandemic. At this point, we are uncertain whether this pattern will persist, and it merits further investigation with additional data from upcoming years.

Many social determinants of health have been shown to contribute to health care disparities in children with ADHD in the United States, such as race/ethnicity (37–39), insurance coverage (39, 40), and a non-English language spoken at home (41). Studies show significant disparities in ADHD diagnosis and medication treatment among African American and Latino children compared to White children (42, 43). A national birth cohort study also reported that Asian children had the highest odds of receiving no treatment (44). In addition, socioeconomic environment and status significantly impact the prevalence, diagnosis, and treatment of ADHD (45–47). Recognizing the need for urgent action, the American Academy of Pediatrics (AAP) prioritized advancing both the AAP Equity Agenda and mental health care for children in 2021 (48, 49). The National Institute of Mental Health’s most recent strategic plan for research (2021) also highlighted the need to reduce disparities and advance equity in mental health services, aiming to improve treatment outcomes in diverse populations (50).

As a case study, we analyzed the zip codes of different stakeholders and examined the potential impact of socioeconomic status on ADHD stimulant use in LA County. Patients 6 to 26 years old in the highest income quintile (Q5) had the highest prevalence per 100,000 people, which trends downward notably with lower incomes. This profile remains consistent in all three years, independent of the pandemic. This may be explained by the underdiagnosis and undertreatment reported in the disadvantaged population (42). Additionally, as reported by the State of California Office of the Patient Advocate, there is a broad variability among different commercial health plans in terms of providing adequate pharmaceutical care and follow-up visits for children with ADHD (51). The CURES dataset, however, does not include specific commercial health care plan information for further analysis. Although our study is limited to LA County, the results underscore the importance of optimizing evidence-based ADHD management to reduce disparities in stimulant use related to patient socioeconomic status.

Furthermore, our analyses revealed that methylphenidate was the predominant medication prescribed to elementary and adolescent populations, whereas mixed amphetamine salts emerged as the primary stimulant used among late adolescents and young adults. The prodrug, lisdexamfetamine, with minimal misuse potential is commonly dispensed to adolescents (12–17 years) in comparison to other age groups. The real-world dispensing profile identified in our study is consistent with a recent meta-analysis, which supports methylphenidate in children and adolescents, and amphetamines in adults as the preferred first-choice medications for ADHD treatment (52). This study provides corroborative data to support evidence-based pharmacotherapeutic recommendations for the use of ADHD stimulants (9).

One limitation of this study is that the California census data is not broken down by the specific age groups used to analyze the CURES data, which prevents us from calculating percentages based on the population size by age. Another limitation is the lack of a full 2018 CURES data set, which restricted us from obtaining a baseline of new patients in 2019 and making comparisons with the years 2020 and 2021. As CURES is an administrative database, it unfortunately does not contain information on individual patient cases providing less contextual information. A future study using electronic health record (EHR) data with more patient medical information may allow us to study other risk factors associated with stimulant use and concurrent controlled substance use.

## 5 Conclusion

In this study, we analyzed real-world dispensing data of ADHD stimulants among children and young adults (≤ 26 years) in California from 2019 to 2021. Our study revealed a significant decline of patients on stimulants among the younger population (3–17 years) during and following the pandemic. These findings also underscore the differences in patient prevalence related to sex and age, and highlight disparities associated with socioeconomic status. The probability of concurrent use of benzodiazepines and/or opioids with stimulants was higher in females (≤ 26 years) and increased with patient age. Our findings provide valuable real-world data concerning a population that may have been overlooked during the pandemic response.

## Data availability statement

The datasets presented in this article are not readily available because CURES data is only obtainable by written request from the Department of Justice with approval. Requests to access the datasets should be directed to the Department of Justice, datarequests@doj.ca.gov.

## Author contributions

AP: Conceptualization, Data curation, Investigation, Methodology, Project administration, Software¸ Writing – original draft, Writing – review & editing. RC: Writing – review & editing. CR: Software, Validation, Writing – review & editing. RB: Conceptualization, Data curation, Investigation, Methodology, Project administration, Resources, Software¸ Supervision, Validation, Writing – original draft, Writing – review & editing. SY: Conceptualization, Data curation, Funding acquisition, Investigation, Methodology, Project administration, Resources, Software¸ Supervision, Writing – original draft, Writing – review & editing.
