# Supplementary material for: Changes in real-world dispensing of ADHD stimulants in youth from 2019 to 2021 in California
Source: Front Public Health. 2024 Mar 5;12:1302144. doi: 10.3389/fpubh.2024.1302144 (PMC10948562; doi:10.3389/fpubh.2024.1302144)
Supplement: Supplementary file 1 [file Data_Sheet_1.docx]

Supplementary Material

Changes in Real-World Dispensing of ADHD Stimulants in Youth From 2019 to 2021 in California

**Anika Patel ^1^, PharmD, Rishikesh Chavan ^2^, MD, Cyril Rakovski ^3^, PhD, Richard Beuttler ^4*^, PsyD, MS, Sun Yang ^1*^, BPharm, MS, PhD**

^1^Department of Pharmacy Practice, Chapman University School of Pharmacy, Irvine, CA 92618

^2^ Hyundai Cancer Institute, CHOC Children’s Hospital, Orange, CA 92868

^3^Department of Mathematics, School of Computational Sciences, Chapman University, Orange, CA 92866

^4^Chapman University School of Pharmacy, Irvine, CA 92618

*** Correspondence:**

Sun Yang, Department of Pharmacy Practice, Chapman University School of Pharmacy, 9401 Jeronimo Road, Irvine CA, 92618, [syang@chapman.edu], (714) 516-5418.

Richard Beuttler, Chapman University School of Pharmacy, 9401 Jeronimo Road, Irvine CA, 92618, [rbeuttle@chapman.edu], (714) 516-5532.

**Supplemental Figure 1: Annual geographic prevalence of patients on ADHD stimulants in each California county from 2019 to 2021.** The color scales indicate the number of patients dispensed a stimulant prescription per 100,000 people. The darkest colors represent the highest prevalence.


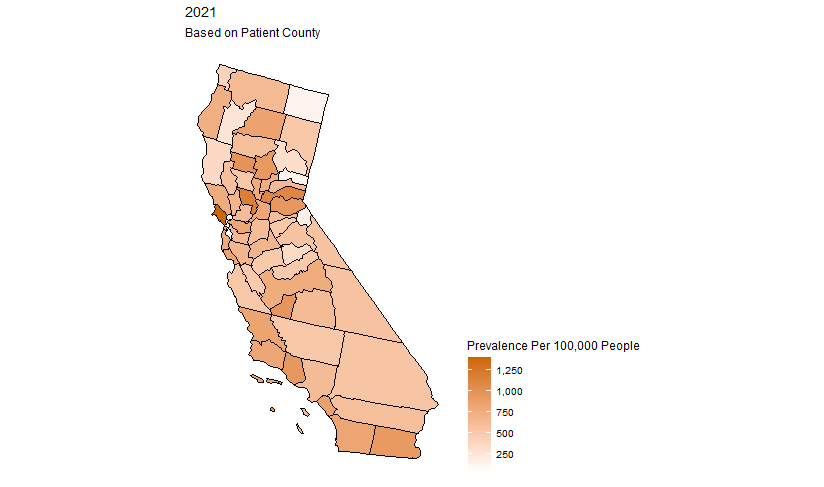
**
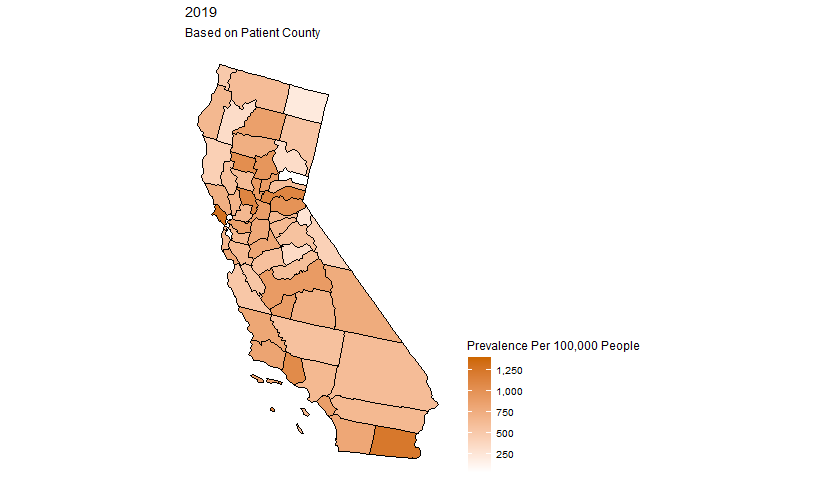

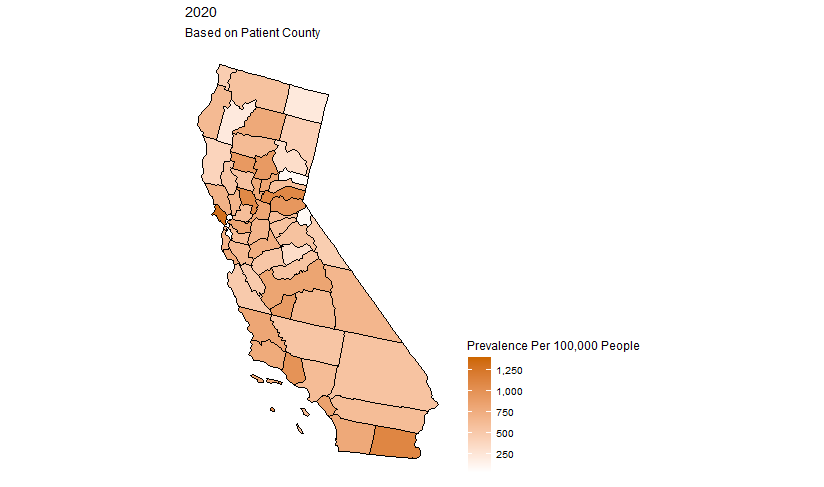

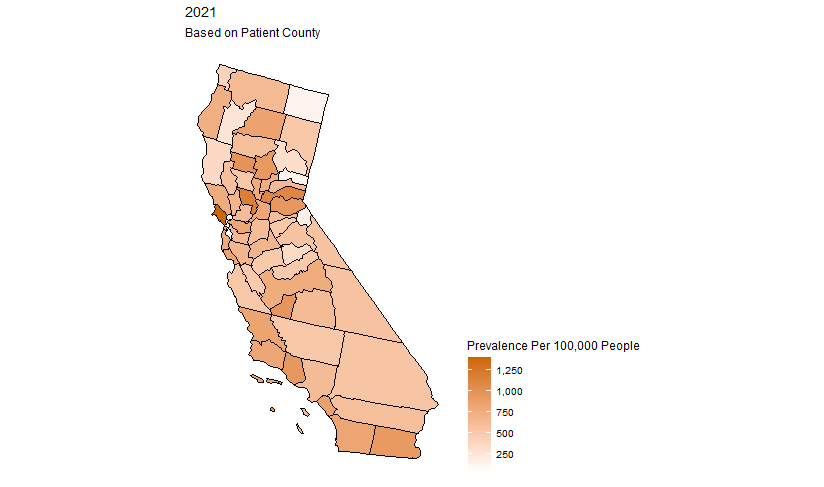
**

**Supplemental Figure 2:** **The sex distribution of patients (3-26 years) on ADHD stimulants by age groups (2019-2021).**

**The number of 0–2-year-old patients was less than 50 in each sex group, which was not included in this figure.*

**Supplemental Figure 3: Patients on concurrent use of ADHD stimulants with benzodiazepines.**

|  | 2019  (n=14 419) (%) | 2020  (n=12 813) (%) | 2021  (n=12 799) (%) |
| --- | --- | --- | --- |
| Sex | | | |
| Male | 6 328 (43.9) | 5 216 (40.7) | 4 809 (37.6) |
| Female | 8 089 (56.1) | 7 595 (59.3) | 7 988 (62.4) |
| Unknown | 2 (<0.1) | 2 (<0.1) | 2 (<0.1) |
| Age Group | | | |
| Infancy (0-2) | 1 (<0.1) | 4 (<0.1) | 1 (<0.1) |
| Preschool (3-5) | 19 (0.1) | 18 (0.1) | 18 (0.1) |
| Elementary (6-11) | 632 (4.4) | 492 (3.8) | 565 (4.4) |
| Adolescent (12-17) | 2 126 (14.7) | 1 700 (13.3) | 1 927 (15.1) |
| Late adolescent (18-21) | 3 169 (22.0) | 2 815 (22) | 2 818 (22.0) |
| Young adults (22-26) | 8 472 (58.8) | 7 784 (60.8) | 7 470 (58.4) |

**Supplemental Figure 4: Patients on concurrent use of ADHD stimulants with opioids.**

|  | 2019  (n=15 341) (%) | 2020  (n=13 635) (%) | 2021  (n=15 257) (%) |
| --- | --- | --- | --- |
| Sex | | | |
| Male | 8 239 (53.7) | 6 823 (50.0) | 7 103 (46.6) |
| Female | 7 091 (46.2) | 6 807 (49.9) | 8 142 (53.4) |
| Unknown | 11 (0.1) | 5 (<0.1) | 12 (0.1) |
| Age Group | | | |
| Infancy (0-2) | 5 (<0.1) | 3 (<0.1) | 5 (<0.1) |
| Preschool (3-5) | 14 (0.1) | 7 (0.1) | 13 (0.1) |
| Elementary (6-11) | 526 (3.4) | 363 (2.7) | 357 (2.3) |
| Adolescent (12-17) | 4 411 (28.8) | 3 696 (27.1) | 3 716 (24.4) |
| Late adolescent (18-21) | 4 656 (30.4) | 4 350 (31.9) | 4 946 (32.4) |
| Young adults (22-26) | 5 729 (37.3) | 5 216 (38.3) | 6 220 (40.8) |

**Supplemental Figure 5: Concurrent benzodiazepine (A) or opioid (B) use with ADHD stimulants among patients ≤26 years in California.** Probability curves show the probability of concurrent controlled substance use based on patient age in 2019 and 2020. Females (red line) showed an increased risk at any age for concurrent use of benzodiazepines or opioids with stimulants in comparison to males (blue line).


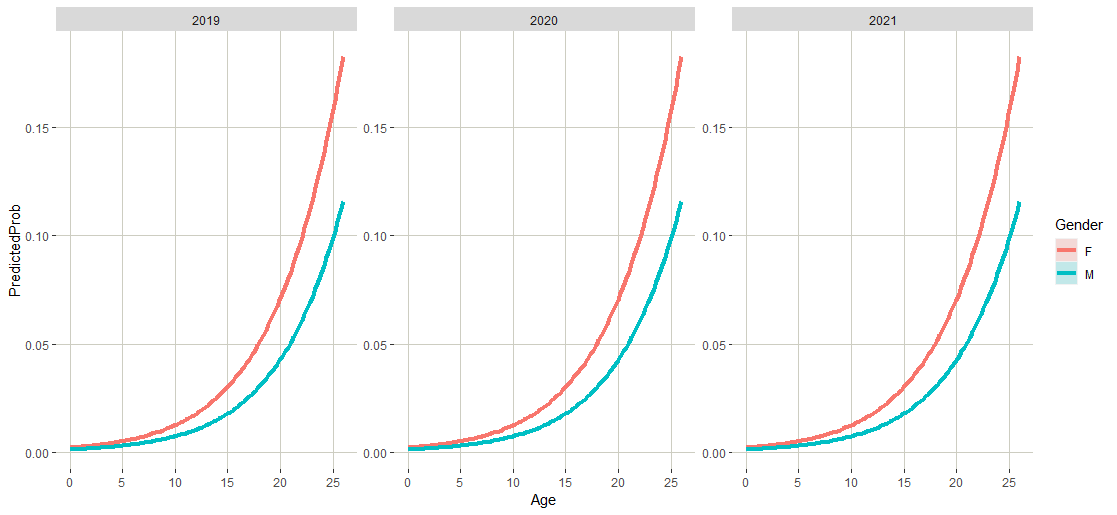

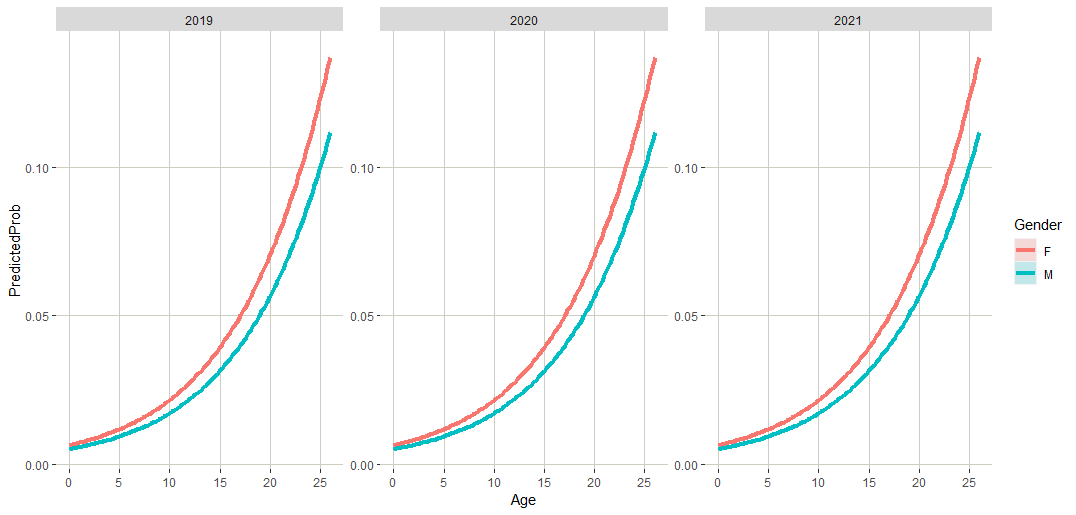


**A**


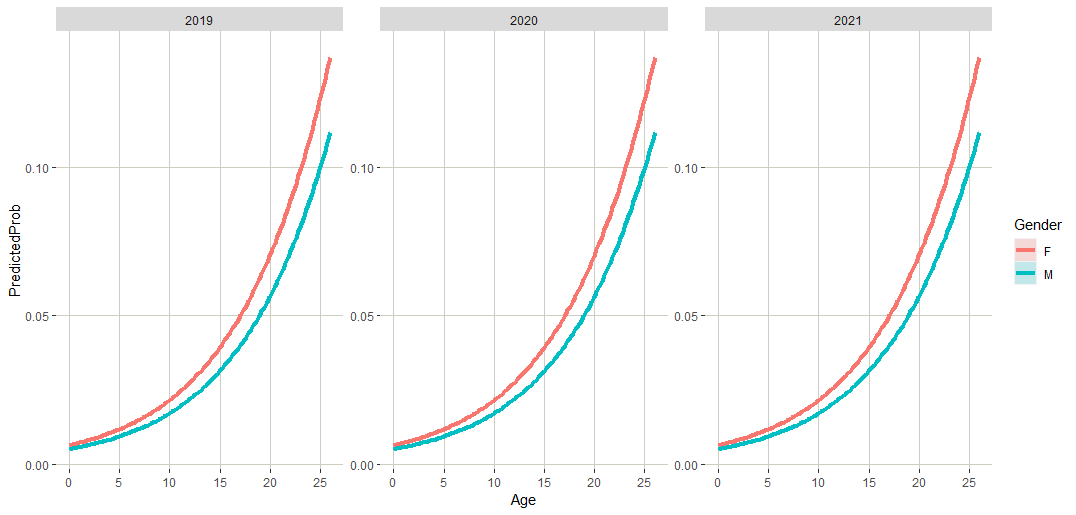

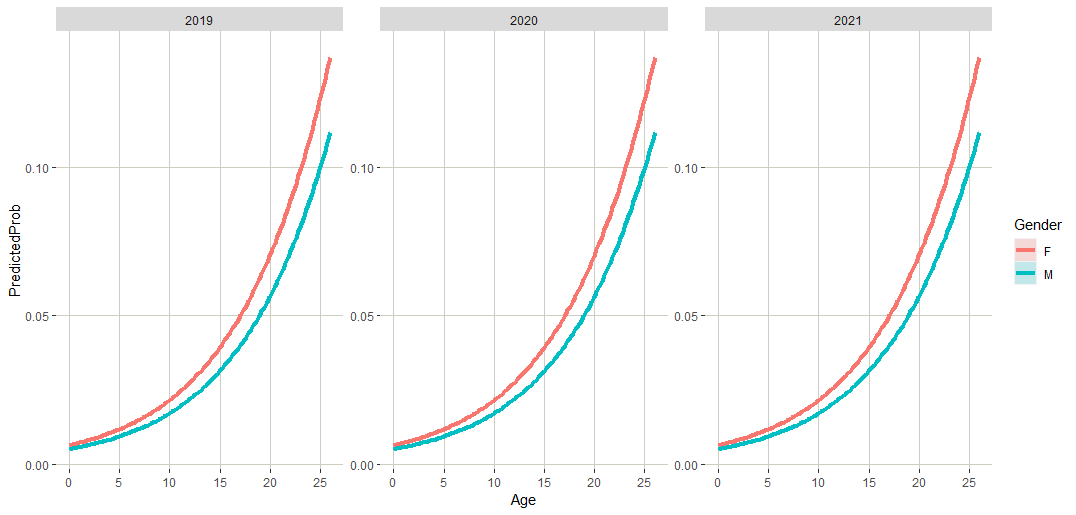


**B**

**Supplemental Figure 6: Trends in ADHD stimulant dispensing based on drug class from 2019 to 2021 in California.** Changes in the number of prescriptions in each class of stimulant medications. Mixed amphetamine salts and methylphenidate are consistently the most dispensed stimulants from 2019-2021.
